# Psoriasis and associated variables in classification and outcome of juvenile idiopathic arthritis - an eight-year follow-up study

**DOI:** 10.1186/s12969-017-0145-5

**Published:** 2017-02-22

**Authors:** Maria Ekelund, Kristiina Aalto, Anders Fasth, Troels Herlin, Susan Nielsen, Ellen Nordal, Suvi Peltoniemi, Marite Rygg, Marek Zak, Lillemor Berntson, on behalf of the Nordic Study Group of Pediatric Rheumatology (NoSPeR), Gudmund Marhaug, Freddy Karup Pedersen, Pirkko Pelkonen, Boel Anderson-Gäre

**Affiliations:** 1 Department of Pediatrics, Ryhov County Hospital, Jönköping, Sweden; 2 Department of Women’s and Children’s Health, Uppsala University, Uppsala, Sweden; 3 Department of Pediatrics, Children’s Hospital, Helsinki University Hospital, Helsinki, Finland; 4 Department of Pediatrics, Institute of Clinical Sciences, Sahlgrenska Academy, University of Gothenburg, Gothenburg, Sweden; 5 Department of Pediatrics, Århus University Hospital, Århus, Denmark; 6 Pediatric Rheumatology Department, Pediatric Clinic II, Copenhagen University Hospital, Rigshospitalet, Copenhagen, Denmark; 7 Department of Pediatrics, University Hospital of North Norway, Tromsø, Norway; 8 Department of Clinical Medicine, UIT The Arctic University of Norway, Tromsø, Norway; 9 Department of Laboratory Medicine, Children’s and Women’s Health, Norwegian University of Science and Technology, Trondheim, Norway; 10 Department of Pediatrics, St. Olav’s Hospital, Trondheim, Norway

**Keywords:** Arthritis, Juvenile Rheumatoid, Psoriasis, Child

## Abstract

**Background:**

To study the impact of psoriasis and features associated with psoriasis on classification and outcome in a population-based follow-up cohort of children with juvenile idiopathic arthritis (JIA).

**Methods:**

In all, 440 children with JIA were followed for a median of 8 years in a prospective Nordic population-based cohort study. Data for remission was available for 427 of these children. The presence of psoriasis, psoriasis-like rash, dactylitis, nail pitting, enthesitis, tenosynovitis and heredity was assessed in relation to ILAR classification and remission.

**Results:**

Clinical findings associated with psoriasis developed consecutively during the 8-year period. Six of 14 children with psoriasis were not classified as juvenile psoriatic arthritis according to the ILAR criteria at 8 year follow-up. Dactylitis was more common in children with early onset of JIA. After 8 years we found a cumulative median number of eleven arthritic joints in children with psoriasis or psoriasis-like rash compared with six in the rest of the cohort (*p* = 0.02). Also, the chance for not being in remission after 8 years increased significantly in patients with psoriasis, psoriasis-like rash or at least two of: 1) first-degree heredity for psoriasis or psoriatic arthritis, 2) dactylitis or 3) nail pitting, compared with the rest of the group (OR 3.32, *p* = 0.010).

**Conclusions:**

Our results indicate a more severe disease over time in psoriasis-associated JIA, as features of psoriasis develop during the disease course. This group is a major challenge to encompass in a future JIA classification in order to facilitate early tailored treatment.

## Background

Classification of children with JIA and features of psoriasis has challenged the research community for decades. The disease comprises a variety of features often developing over the years, with large heterogeneity. Since classification criteria have varied over time there have also been dissimilarities in the cohorts studied. The question has been raised whether juvenile psoriatic arthritis (JPsA) is an entity of its own [1] or whether psoriasis and juvenile arthritis develop only as separate entities [2–4]. Adult psoriatic arthritis (PsA) is considered a unique disease entity, clearly distinct from rheumatoid arthritis. Immunology research has revealed that molecules of the interleukin-23 and interleukin-17 pathways promote skin and joint inflammation in psoriasis [5]. These discoveries have led to efforts to target interleukin-23, interleukin-17 and related molecules to treat psoriasis and psoriatic arthritis [6].

Classification of JPsA has been a challenge. In 1989, the Vancouver criteria for JPsA were presented comprising two categories of JPsA, one definite and one probable [7]. In three studies utilizing the Vancouver classification criteria, joint patterns differed at onset between oligoarticular JIA and oligoarticular JPsA, with greater involvement of small joints in the hands and wrists being more typical in the latter category [1, 8, 9]. Age at onset was significantly higher in oligoarticular JPsA compared with oligoarticular JIA. A child with an oligoarticular pattern with wrists or small joints in hands or feet affected, more often had nail pitting, psoriasis or heredity for psoriasis.

In the current ILAR classification, JPsA may be diagnosed in children with arthritis and (1) psoriasis or (2) if two features suggestive of a psoriatic diathesis are present, including dactylitis, nail changes, or psoriasis in a first-degree relative, in the absence of specific exclusions [10].

During later years two distinct phenotypic subgroups of JPsA have been discussed. One occurs in children (predominantly girls) younger than 5 years of age at onset, with small joint involvement, a higher risk of progression to polyarticular disease, high frequency of ANA positivity and dactylitis. In contrast, children above 5 years of age at onset had no obvious gender difference, were more likely to be ANA negative and to exhibit enthesitis and axial joint involvement [7, 11, 12].

So far, the prognosis for JPsA has been considered approximately the same as for patients with other categories of JIA based on two follow-up studies, one using both the Vancouver and the ILAR criteria with a follow-up time of 1 month, up to 16.8 years [2] and the second using the Vancouver criteria with a median follow-up time of 2 years [13]. Children with a polyarticular course had the worst prognosis. Positive ANA in children with JPsA has been discussed as a negative prognostic factor without any conclusive results [7, 9, 14]. Occurrence of HLA-B27 has been shown as a risk factor for sacroiliitis in JPsA [2].

In light of this knowledge, we wanted to challenge earlier findings by studying a well-defined cohort of children with JIA, followed prospectively over a long time, especially since previous studies were referral-based. Our aim was to study the occurrence of variables indicating psoriatic diathesis during the first 8 years of disease in a population-based cohort of children with JIA and to address aspects of classification and outcome.

## Methods

Patients were recruited from a Nordic JIA cohort. All new patients with JIA from defined geographical areas of Denmark, Finland, Norway, and Sweden were collected prospectively during 1997–2000 [15]. The study design was population-based. All physicians within each catchment area eligible to meet children with arthritis received a letter informing about the study. Pediatric rheumatologists from 12 participating centers included patients. The database consisted of clinical and laboratory data from 500 patients, including 440 followed for at least 7 years, median 98 months. The 60 patients lost to follow-up did not differ from the 440 with follow-up data as regards number of active joints during the first 6 months after onset, Childhood Health Assessment Questionnaire (CHAQ), Juvenile Arthritis Disease Activity Score (JADAS27), or proportion with oligoarticular disease at baseline. Also, occurrence of psoriasis, psoriatic-like rash, dactylitis, enthesitis, first degree heredity for psoriasis or psoriatic arthritis did not differ between the 60 lost to follow-up compared to the 440 in the study cohort. JIA categories had been determined based on the ILAR criteria [10]. For 427 of 440 children, outcome data was available for analysis of remission. The presence of psoriasis and psoriasis-like rash during the 8-year follow-up period was assessed in relation to clinical characteristics, ILAR classification and remission. Remission status according to the preliminary criteria of Wallace et al was used [16]. Clinical variables were collected cumulatively during this period; they were not necessarily present at the time of the 8-year follow-up. A patient was considered to have psoriasis if a dermatologist had made that diagnosis. Rashes noted by the examining rheumatologist, well skilled in pediatric rheumatology, and thought likely (but not definitely) to represent psoriasis were considered psoriasis-like.

## Results

In the cohort of 440 children followed for at least 7 years after disease onset (median 98 months, range 84–147 months), 14 children developed psoriasis and 13 psoriasis-like rash (Table 1). Only 8 of the 14 children with psoriasis and JIA were classified as juvenile psoriatic arthritis (JPsA) according to the ILAR criteria. The other six were classified as undifferentiated. Four of those were mutually excluded from JPsA as well as enthesitis related arthritis (ERA), thus they did not fit any category. One boy was excluded because he was older than six at onset and HLA-B27 positive, and another child was excluded because of positive rheumatoid factor (RF). Children with dactylitis and/or nail pitting were found among many ILAR categories. First-degree heredity for psoriasis or psoriatic arthritis occurred in 9 of 14 children with JPsA and in 51 of 57 of children classified as undifferentiated arthritis. In a comparison between the eight children with psoriasis, classified as JPsA (ILAR criteria) at 98 months of disease, and the six children with psoriasis classified as undifferentiated, there was no significant difference in age at onset (*p* = 0.34), first degree heredity for psoriasis/psoriatic arthritis (*p* = 0.53), dactylitis (*p* = 1.0), nail pitting (*p* = 0.30) or cumulative number of joints (*p* = 0.59), but the cohorts were too small for meaningful statistical analyses. Children with psoriasis (*n* = 14) compared to those with psoriatic-like rash (*n* = 13) did not differ regarding gender (*p* = 0.71), outcome (*p* = 0.16) or median cumulative number of joints (*p* = 0.26).

**Table 1:** Clinical characteristics in a Nordic cohort of children with juvenile idiopathic arthritis, classified according to the ILAR classification criteria^b^ and followed for at least seven years

Clinical characteristics								
	Number of patients n	Age at onset median (IQR)	Psoriasis n	Psoriasis- like rash n	Dactylitis n	Nail pitting n	Patients assessed for heredity n	First-degree heredity for psoriasis or psoriatic arthritis n (%)
Systemic arthritis	18	4.5 (2.2-6.7)	-	-	-	-	18	-
Oligoarticular persistent	133	5.1 (2.6-8.5)	-	1	6	3	123	-
Oligoarticular extended	78	4.1 (1.8-8.4)	-	2	1	3	74	-
Polyarticular RF negative	80	4.9 (2.2-9.1)	-	2	7	-	79	-
Polyarticular RF positive	3	10.2, 13.2, 13.3	-	-	-	-	3	-
Psoriatic arthritis	14	5.8 (4.3-7.9)	8	3	3	7	14	9 (64.3)
Enthesitis-related arthritis	49	10.0 (6.8-12.3)	-	3	1	2	47	-
Undifferentiated arthritis	65	7.8 (3.2-11.8)	6^a^	2	6	3	57	51 (78.5)

^a^None of the six patients with psoriasis at the 8-year follow-up fit any category, four because of mutual exclusion from JPsA and ERA because of enthesitis, one because he was a HLA-B27-positive boy older than 6 years at onset, one because of positive RF

^b^International League of Associations for Rheumatology classification criteria [10] assessed at last study visit (median 98, range 84–147 months)

Table 2 presents the development of clinical features associated with psoriasis during the first 8 years of disease. Twenty-four children developed dactylitis during disease course, 15 of them had dactylitis at onset. Median age at time of onset of JIA in the group with dactylitis was 2.4 (IQR 1.7–5.4) years compared with 5.8 (IQR 2.7–9.9) years in the rest of the cohort (*p* = 0.007).

**Table 2:** Development of clinical features associated with psoriasis in 440 children with JIA followed for at least seven years after onset (median 98, range 84–147 months)

Clinical features	Patients at onset n	Cumulative number of patients at eight years n
Dactylitis	15	24
Nail pitting	10	18
Psoriasis	11	14
Psoriasis-like rash	6	13
Enthesitis	37	41

Five of 24 children with dactylitis developed psoriasis or psoriasis-like rash during the first 8 years of their arthritis, Table 3. Table 3 also presents the significantly higher cumulative number of active joints in children with psoriasis or psoriasis-like rash as well as the increased frequency of dactylitis, nail pitting, enthesitis, and first-degree heredity for psoriasis compared with children without psoriasis or psoriasis-like rash.

**Table 3:** Clinical features describing the first eight years of disease in 440 children with JIA according to occurrence of psoriasis or psoriasis-like rash

Clinical feature	Number of patients assessed	Total cohort	Psoriasis or psoriasis-like rash *n* = 27	No psoriasis or psoriasis-like rash *n* = 413	*p*-value^*^
Gender, n (% females)	440 (66.1)	440 (66.1)	27 (48.1)	413 (67.3)	0.06^a^
Age at time of onset median (IQR)	440	5.5 (2.5-9.7)	7.2 (3.8-11.0)	5.3 (2.3-9.4)	0.08^b^
Cumulative joints median (IQR)	440	6 (2-12)	11 (5-16)	6 (2-12)	0.02^c^
Dactylitis (%)	440	24	5 (18.5)	19 (4.6)	0.01^a^
Nail pitting (%)	440	18	7 (25.9)	11 (2.7)	<0.001^a^
First-degree heredity for psoriasis or psoriatic arthritis (%)	420	51	7 (25.9)	44 (10.6)	0.03^a^
Enthesitis (%)	438	41	6 (22.2)	35 (8.5)	0.03^a^
Tenosynovitis (%)	436	88	6 (22.2)	82 (19.8)	0.81^a^

^*^Patients with psoriasis or psoriasis-like rash compared with those without, ^a^Fisher’s exact test, ^b^Independent samples Mann-Whitney *U* test, ^c^Independent samples median test

Features of sacroiliitis (SI) developed in several children with psoriasis or psoriasis- like rash (Table 4). SI or enthesitis coexisting with psoriasis or psoriasis-like rash lead to mutual exclusion from both JPsA and ERA categories.

**Table 4:** Development of clinical variables during a follow-up time of at least seven years after onset (median 98, range 84–147 months) in groups of patients included in a cohort of 440 Nordic patients with JIA

	Psoriasis or psoriasis-like rash		Dactylitis	
	*n* = 27	*n* ^a^	*n* = 24	*n* ^a^
Radiological SI^b^				
At onset		0		
Cumulative at median 98 months		2		0
Inflammatory back pain/buttock pain				
At onset		3		0
Cumulative at median 98 months		6		0
Enthesitis				
At onset		5		3
Cumulative at median 98 months		6		4

^a^Number of patients with the feature developing during the first median 98 months

^b^ *SI* sacroiliitis

Seven of 27 children with psoriasis or psoriasis-like rash were HLA-B27 positive (missing value in another three) and this group had a high age of onset (median 10.8, IQR 5.9–11.8 years), and a high cumulative number of joints (median 15, IQR 9–18 joints), data not shown. Several of the ERA or SI-like features were clustered together. Six of the 27 children with psoriasis or psoriasis-like rash had inflammatory back pain or buttock pain at any time during the first 98 months of disease (Table 4), three of those were HLA-B27 positive, and in two sacroiliitis was confirmed by x-ray.

In children with psoriasis or psoriasis-like rash or at least two of either dactylitis, nail pitting, or first-degree heredity of psoriasis, the remission rate was significantly lower compared with children without these clinical features, *p* = 0.010 (Table 5).

**Table 5:** Analyses of binary logistic regression for not being in remission related to psoriasis and psoriasis-related variables in 427 children with JIA, followed for at least seven years of disease

		Number of patients	Not in remission^c^after 8 years n (%)	*p*-value	OR	95% confidence interval
Psoriasis or psoriasis-like rash^a^	Yes	25	19 (76.0)	0.062	2.44	0.96-6.2
	No	402	227 (56.5)			
Psoriasis or psoriasis-like rash OR at least two of three^b^	Yes	31	25 (80.6)	0.010	3.32	1.33-8.27
	No	379	211 (55.7)			
Dactylitis	Yes	24	16 (66.7)	0.36	1.5	0.63-3.6
	No	403	230 (57.1)			
Nail pitting	Yes	18	13 (72.2)	0.21	1.96	0.69-5.6
	No	409	233 (57.0)			
Enthesitis	Yes	40	29 (72.5)	0.046	2.08	1.01-4.29
	No	385	215 (55.8)			
First-degree heredity for psoriasis or psoriatic arthritis	Yes	48	28 (58.3)	0.86	1.1	0.42-2.82
	No	361	208 (57.6)			
Total		427	181 (42.4)			

^a^Data in 13 patients with psoriasis and 12 with psoriasis-like rash

^b^1) dactylitis, 2) nail pitting, 3) first-degree heredity for psoriasis or psoriatic arthritis

^c^Remission was defined as inactive disease off medication for at least twelve months in accordance with the preliminary definition of Wallace [16]

First-degree heredity for psoriasis in children with oligoarticular onset did not increase the risk for oligoarticular extended disease during those first 8 years of disease (data not shown).

### Statistical analysis

Statistical analyses were performed using the Statistical Package for Social Sciences, version 23 (SPSS Inc., Chicago, IL, USA). Demographics and disease characteristics were described using median and interquartile range (IQR), or total number and percent of study cohort. Statistical analyses of differences between children with and without psoriasis/psoriasis-like rash during the first year of disease were performed using the chi-square test (Fisher’s exact test, 2-sided) for comparison of dichotomous variables. The Mann-Whitney *U*-test was used for comparison of non-parametric data and independent samples was used for comparison of median values between the two groups.

Binary logistic regression analyses were performed in order to identify the association between occurrence of psoriasis or psoriasis-like rash and failure of remission 8 years after disease onset. The dichotomized variable remission (remission without medication) versus remission with medication or not in remission was used as the dependent variable in the regression model. Results of the regression analyses are shown as odds ratios (OR), 95% confidence intervals (CI) and *p*-values. The level of significance was set at 5% (*p* < 0.05).

## Discussion

In this population-based study of 440 patients followed for a median of 98 months, we found that as many as six of 14 children with definite psoriasis were classified as undifferentiated JIA based on exclusion criteria in the ILAR classification. In children with psoriasis or psoriasis-like rash we found a strong association with first-degree heredity for psoriasis or psoriatic arthritis, dactylitis and nail pitting, but also with enthesitis. Psoriasis or psoriasis-like rash was also associated with higher cumulative number of active joints and possibly with less favorable outcome, as features of psoriasis are additive.

A strength of our study was the prospective population-based design and also that the whole cohort was followed for at least 7 years. A weakness was that the group of children with psoriasis or psoriasis-like rash was small and no dermatologist verified the psoriasis-like rash. The pediatric rheumatologists involved in the study all had several years’ experience and a psoriasis-like rash was only defined if there was a high suspicion of psoriasis.

Another clinical sign, dactylitis, has proven a strong predictor of JPsA [13]. In preparative studies for classification of psoriatic arthritis in adults (CASPAR criteria), the specificity of dactylitis was shown to be 95%, nail dystrophy 98% and tender enthesitis and diffuse enthesitis pain 77–93% [17]. The strong association between psoriatic arthritis, dactylitis, nail changes and enthesitis is in accordance with our results. In our study cohort occurrence of dactylitis was spread among ILAR categories, but had a strong association with psoriasis or psoriasis-like rash. Also, enthesitis showed a strong association with psoriasis or psoriasis-like rash in our study cohort. Although enthesitis is a well-recognized feature of PsA in adults, it is an exclusion criterion for JPsA in the ILAR classification, and a common reason for exclusion from the JPsA category [15]. Another important feature in psoriatic diathesis in adults is heredity for psoriasis [17] and our results pointed in the same direction.

In our cohort, first-degree heredity for psoriasis was common in the undifferentiated JIA category, which has also been discussed by others and illustrates the classification difficulties [18]. Stoll et al studied classification of juvenile psoriatic arthritis and found that the ILAR criteria excluded almost 60% of those included with the Vancouver criteria [19], which is in line with our results regarding ILAR classification.

Another challenge in classification is the progression of the disease over time. In an earlier publication we showed that patients classified as JPsA according to the ILAR criteria increased from 6 to 14 during the follow-up period. In about half of the children with JPsA, the classic rash presented after the onset of arthritis, with a time lag that could be 10 years or more [20]. Manifestations of psoriasis in the young child are often atypical or unspecific, such as erythema and scaling behind the ears, features taken into consideration by the Vancouver set of JPsA criteria, but excluded under the JIA nomenclature in the ILAR classification [7]. Our data presents the heterogeneity of JPsA and the development over time of clinical variables supporting a psoriatic diathesis, as well as the overlap between JPsA and enthesitis-related arthritis in a group of patients.

The earlier findings of two phenotypic subgroups of JPsA: a younger group, predominantly girls, with a polyarticular onset, high frequency of ANA positivity and dactylitis [13], in contrast to an older age group more likely to exhibit enthesitis and axial joint involvement [7, 13], can only partly be confirmed by our results since our cohort was too small for further analysis. We found a significantly lower age at time of onset in children who developed dactylitis during the 8 years, compared with those who did not. None of the 15 children with dactylitis at disease onset developed inflammatory back pain during the disease course and in the extended group of 24 children with dactylitis after 8 years, none developed any sign of sacroiliitis. This result is consistent with a previous observation that patients with dactylitis and sacroiliitis tend to be distinctive phenotypic groups of juvenile psoriatic arthritis [21]. Our finding of an older age at onset in the group of HLA-B27 positive children with psoriasis or psoriasis-like rash also supports earlier data, but we were surprised by the very high cumulative number of joints with arthritis in this group.

The polyarticular disease progression in children with psoriasis and arthritis in our study is in line with earlier studies [7, 9, 12]. Our result indicates a somewhat less favorable outcome in the cohort of children with psoriasis, psoriasis-like rash or at least two well-known features characterizing the JPsA group, but our numbers are too low to analyze each of the clinical features as a single trait, and larger studies are needed to confirm our results. The prognosis in JPsA defined according to the Vancouver classification criteria was not worse compared with other JIA categories in earlier studies [2, 13], but the time of follow-up varied: 1 month in one study up to 17 years, and a median of 23 months in the other. By comparison, our cohort was followed for at least 7 years, with a median of 98 months, and in a population-based setting.

Since classification of JPsA has changed over time, it is difficult to compare one study with another and to properly present outcomes in this group of patients. In our study clinical findings associated with psoriasis as well as first-degree heredity for psoriasis were strongly associated with children developing psoriasis or psoriasis-like rash during the first 8 years of disease. Our cohort also reveals the difficulty of using the ILAR classification to assign those children. The consequence is that we do not study children with a psoriatic diathesis specifically, which would be important in relation to new therapies under development for JPsA and bringing these children new treatment possibilities.

## Conclusions

We suggest stronger emphasis on the variables of psoriatic diathesis, and not using strict exclusion criteria in future follow-up studies of JIA, and to consider this also in future JIA classifications. We need to better understand how to predict this category of disease and to be able to tailor proper treatment for this group of children.

## Abbreviations

CHAQ: Childhood health assessment questionnaire
CI: Confidence interval
ERA: Enthesitis related arthritis
ILAR: The international league of associations for rheumatology
IQR: Interquartile range
JADAS27: Juvenile arthritis disease activity score
JIA: Juvenile idiopathic arthritis
JPsA: Juvenile psoriatic arthritis
PsA: Psoriatic arthritis
RF: Rheumatoid factor
